# Characterization of *Pseudomonas aeruginosa* with discrepant carbapenem susceptibility profile

**DOI:** 10.1186/s12941-016-0127-3

**Published:** 2016-02-24

**Authors:** Agila K. Pragasam, M. Raghanivedha, Shalini Anandan, Balaji Veeraraghavan

**Affiliations:** Department of Clinical Microbiology, Christian Medical College, Vellore, 632004 India

**Keywords:** Carbapenems, *Pseudomonas aeruginosa*, Porins, Efflux

## Abstract

*Pseudomonas aeruginosa* is the most common nosocomial pathogen, notorious for its multidrug resistance and causes life threatening infections. Carbapenems were considered as the last resort of drugs for the treatment of multi drug resistant *P. aeruginosa* infections. The emergence of resistance to carbapenems limits its use for treatment. Unlike other organisms, in *P. aeruginosa* intrinsic/chromosomal mediated resistance mechanisms plays a major role for carbapenem resistance rather than the carbapenemases. Carbapenemase producing organisms becomes resistant to both imipenem and meropenem. However, in our clinical settings, we have observed rare carbapenem resistant phenotypes such as imipenem resistant but meropenem susceptible (IRMS) and meropenem resistant but imipenem susceptible (MRIS) phenotypes. Thus we have chosen these rare phenotypes to look for the respective resistance mechanisms by phenotypic and molecular methods. From this study we found that, IRMS is primarily due to the mutations across various regions in the loops of *oprD* gene and MRIS is due to the over expression of *mexAB* efflux pumps. This study results confirms that, this rare phenotypes are due to the intrinsic/chromosomal mediated mechanisms, which occurred due to the antibiotic selection pressure. This study also provided data concerning alterations in outer membrane permeability which is often associated with the increased levels of antibiotic efflux. Consequently, this study provided the prevalence of the various resistance mechanisms that have deployed by the organism to resist antibiotics through different phenotypes.

*Pseudomonas aeruginosa* is a nosocomial pathogen which can cause a wide range of infections in humans, especially in immunocompromised patients. *P. aeruginosa* has intrinsic resistance against most of the antipseudomonal drugs. Carbapenems are considered to be the drug of choice for the treatment of infections due to multi drug resistant *P. aeruginosa.* However, due to the inappropriate drug use and improper dosage of carbapenem, development of drug resistance can occur in clinical settings. Among the carbapenems, imipenem is the least preferable drug, due to the high dose therapy with MIC of 4 µg/ml, whereas doripenem have an edge over meropenem and imipenem [1]. Unlike carbapenemase mediated resistance in *Enterobacteriaceae*, intrinsic mediated resistance mechanisms play a significant role for carbapenem resistance in *P. aeruginosa*. Mechanisms associated with carbapenem resistance can be classified as plasmid mediated and chromosomal mediated. Plasmid mediated are the carbapenemase enzymes which can hydrolyze imipenem and meropenem; but doripenem to a lesser extent [2]. On the other hand, chromosomal mediated mechanism are specific for each carbapenems, such as, loss of porin (*oprD*) contributing to imipenem resistance and the overexpression of efflux pumps contributing to meropenem resistance [3]. This is due to the target specific uptake and pumping out of carbapenems because of the structural variations. Clinical isolates that express either of these mechanisms contributes to different types of phenotypes such as type I (imipenem resistant meropenem susceptible—IRMS), type II (meropenem resistant imipenem susceptible—MRIS) and type III (imipenem resistant meropenem resistant—IRMR). To understand the resistance mechanisms present among the *P. aeruginosa*, we have chosen isolates that shows discrepancy in the carbapenem susceptibility pattern. These isolates were tested for various resistance mechanisms that contribute for such rare phenotype.

Generally, in our experience, isolates that harbor any of the plasmid mediated carbapenemases gene gives rise to type III (IRMR) phenotype of *P. aeruginosa.* However, we observed, type I (IRMS) and type II (MRIS) clinical isolates of *P. aeruginosa* in our clinical settings. There is very little information available on these rare phenotypes, this study was carried out to characterize their respective resistance mechanisms responsible for such rare phenotypes. Based on the carbapenem susceptibility patterns, a total of seven IRMS and three MRIS isolates that showed discrepancy in the carbapenem susceptibility were chosen for the study. All these isolates were isolated from blood and sputum samples between July and December 2014 from patients admitted in Christian Medical College, Vellore. The test isolates were identified up to species level as a part of routine diagnostic cultural identification methods and antimicrobial susceptibility testing was done by Kirby Bauer disc diffusion method, minimum inhibitory concentration (MIC) was determined for imipenem, meropenem and doripenem by E-test method (BioMerieux) and interpreted according to CLSI guidelines [4]. In addition, all the ten isolates were subjected to classical CarbaNP test using BPER-II lysis protocol [5], modified hodge test according to CLSI guidelines [CLSI M100-S21], broth micro dilution using PAβN as an efflux pump inhibitor (at 20 µg/ml) in combination with levofloxacin and multiplex PCR for the detection of *bla*_IMP_ [6]*, bla*_VIM_ [7]*, bla*_NDM_ [8], *bla*_KPC_*[*9*]* and *bla*_Oxa-48 like_ [10] genes. Relative quantification of *oprD* transcripts and *mexAB* efflux pumps was done by RT-qPCR [11] for IRMS and MRIS isolates respectively as per the described methods. In addition, *OprD* gene was sequenced for all the seven isolates of IRMS *P. aeruginosa* at Christian Medical College, Vellore. (ABI Prism 3100 Genetic Analyzer—Applied Biosystems) and also multi locus sequence typing (MLST) was done as described previously [12].

The MICs of imipenem, meropenem and doripenem were given in Table 1. CarbaNP and modified hodge test showed all the isolates were negative, indicating the absence of carbapenemases. Subsequently, multiplex PCR for the detection of *bla*_IMP_*, bla*_VIM_*, bla*_NDM_*, bla*_KPC_ and *bla*_Oxa-48 like_ genes were also negative and the results are mentioned in Table 1. This strongly indicates that the resistance mechanisms responsible for type I and type II susceptibility pattern were not due to the plasmid mediated carbapenemases. Furthermore, upon sequencing of *oprD* gene in IRMS isolates, many different mutations were observed across various loops in the *oprD* porin. Relative quantification of *oprD* transcripts revealed that, out of 7 IRMS isolates, porin down regulation was observed in 3 of the isolates, while the other 4 isolates had basal level expression. On the other hand, MRIS isolates showed over expression of *mexAB* efflux pumps and the results are mentioned in Table 2.

**Table 1:** Phenotypic and molecular characterization results of IRMS and MRIS *P. aeruginosa* isolates

Micro No.	Imipenem MIC (µg/ml)	Meropenem MIC (µg/ml)	Doripenem MIC (µg/ml)	CarbaNP test	MHT	Multiple × PCR^a^	*oprD* mutations	RT-qPCR *oprD* (fold difference)	RT-qPCR *mexAB* (fold difference)	Sequence Types-ST (MLST)
Imipenem resistant meropenem susceptible *P. aeruginosa* (IRMS)										
PA3970	4	4	4	Negative	Negative	Negative	L4, L6	2.31	–	–
PA5778	6	1.5	1	Negative	Negative	Negative	L4, L6	0.81	–	ST 1685
PA4745	>32	4	2	Negative	Negative	Negative	L2, L7, L8, L9, L15	Undetermined	–	–
PA6192	4	2	0.5	Negative	Negative	Negative	L2, L7, L8, L9, L15	0.16	–	ST 1993
PA6362	4	1	1	Negative	Negative	Negative	L4, L6	1.27	–	ST 360
PA6475	32	2	1.5	Negative	Negative	Negative	Mutations in primer binding site	Undetermined	–	ST 1952
PA6476	4	1	1	Negative	Negative	Negative	L2, L7, L8, L9, L15	0.39	–	ST 617
Meropenem resistant imipenem susceptible *P. aeruginosa* (MRIS)										
PA4381	6	4	3	Negative	Negative	Negative	Not done	Not done	3.37	–
PA5350	4	32	4	Negative	Negative	Negative	Not done	Not done	2.54	–
PA26815	3	8	12	Negative	Negative	Negative	Not done	Not done	1.09	–

Genes tested: *blaIMP,blaVIM, blaNDM, blaKPC, blaOXA*-*48 like genes.* Relative quantification of *oprD* and *mexAB* expression was normalized with ATCC 27853 *P. aeruginosa* with a value of 1.0 and the fold difference was mentioned above

**Table 2:** Mutations observed in *oprD* gene of IRMS *P. aeruginosa* isolates

Micro No.	Amino acid changes	Nucleotide changes		Impact on *oprD* loops
		Deletions	Positions	
PA3970	T103S, K115T, F170L	None	None	L4, L6
PA4745	S57E, N262T	180 nts 332 nts	480–660 828–1219	L2, L7, L8, L9, L15
PA5778	K115T, V436D^a^	3 nts 2 nts 1 nt	632–634 656–657 770, 1274, 1278, 1287, 1332	L4, L6
PA6192	S57E,S59R,I210A,N262T,A281G,R310G,G425A “MSDNNVGYKNYG’’ to ‘‘VDSSSSYAGL’’ at position *372–383*	3 nts 4 nts 1nt 1 nt (insertion)	1116–1118 1147–1150 1328 1138	L2, L7, L8, L9, L15 Loss of Porin
PA6476	S57E,S59R,I210A,N262T,A281G,R310G,E426R “MSDNNVGYKNYG’’ to ‘‘VDSSSSYAGL’’ at position *372–383*	3 nts 4 nts 1 nt (insertion)	1116–1118 1146–1149 1138	L2, L7, L8, L9, L15
PA6362	K115T, L443 W	1 nt 1nt	342 1329	L4, L6

^a^Newly observed mutation which was not reported so far

Expression of mRNA transcripts of *oprD* gene and *mexB* gene was analyzed by relative quantification in real time qPCR and the expression of study isolates were normalized with ATCC 27853 *P. aeruginosa* gene expressions which is assigned a value of 1. Among the seven IRMS phenotypes, PA5778, PA6192 and PA6476 showed down regulation of *oprD* which was <1.0 (Table 1). In addition, mutations was observed in; L2, L7, L8 L9 and L15 for PA6192 and PA6476. In PA5778, mutations were observed in L4 and L6 with a mutation of V436D, which was not reported in the literature so far. Whereas for, PA3970 and PA6362, *oprD* expression was not down regulated. However, PA3970 and PA6362 had mutations in loop 4 and loop 6 regions of *oprD*, which had an impact on imipenem resistance in these two isolates. In PA4745 and PA6475, quantification of *oprD* transcripts was failed due to the presence of mutations in the primer binding regions of *oprD* gene. While sequencing of PA4745 *oprD* gene revealed that there was huge deletion of about 612 nucleotides which affects the L2, L7, L8, L9 and L15 regions which is the cause for imipenem resistance. These mutations except V436D were reported in previous studies [13]. Whereas, sequencing of *oprD* gene in PA6475 could not be accomplished due to the mutations in the forward primer binding regions, thus the mutation was not able to be determined by sequencing. Overall, mutations in *oprD* gene play a significant role in contributing resistance to imipenem in these isolates. Moreover, multi locus sequence typing showed that, the isolates of IRMS were not of the same sequence types. The different sequence types were ST1685, ST1993, ST360, ST1952 and ST617. These sequence types of IRMS isolates were previously found in Canada, China, Australia, Senegal, Brazil and The Netherlands (pubmlst.org).

Among three MRIS isolates, phenotypic characterization of efflux pumps with carbenicillin with PAβN showed > fourfold difference for PA4381 and PA5350, which was also correlated well with the relative quantification of *mexAB* pump which showed >1 for *mexB* expression in comparison to the control ATCC 27853 *P. aeruginosa.* This confirms that the meropenem resistance in type II (MRIS) phenotypes was due to the over expression of *mexAB* pump. While one isolate PA26815 showed < fourfold difference in the PAβN combination testing, which was also correlated with the real time quantification where, the *mexAB* expression was similar to ATCC 27853 *P. aeruginosa*. However, while the cause for meropenem resistance is not due to the *mexAB* pump, it could be due to any other resistance mechanism which needs to be studied further.

The emergence of aforementioned phenotypes occurs mainly due to the antibiotic selection pressures promoted by inappropriate dosage and duration of the carbapenems. Importantly, it is advisable to perform antimicrobial susceptibility testing for each of the carbapenems namely imipenem, meropenem and doripenem, rather than testing single agent and extrapolating results for the other carbapenems. Apart from individual testing, another difficulty is in interpreting the results. This is due to the differences in the breakpoint cut off recommended by CLSI [4] and EUCAST [14]. Wherein 2 µg/ml is being susceptible by CLSI and 2 µg/ml being resistant by EUCAST for doripenem. Such dissimilarities complicate the interpretation and use of doripenem which remains as the drug of choice for treating infections due to these phenotypes. It is important to avoid selection pressures which contribute for the emergence and spread of these discrepant phenotypes in clinical settings.
